# Influence of eccentric tempo on concentric performance in competitive Paralympic bench press

**DOI:** 10.3389/fspor.2026.1963961

**Published:** 2026-10-26

**Authors:** Arthur Luz dos Santos, Bárbara G. Silva, Silvio S. Santos, Thiago M. Fidale, Frederico B. Lizardo, Weverton Santos, Felipe J. Aidar, Michal Wilk, Adriano A. Pereira

**Affiliations:** 1 Postgraduate Program in Biomedical Engineering, Federal University of Uberlândia, Uberlândia, Brazil; 2 Faculty of Physical Education and Physiotherapy, Federal University of Uberlândia, Uberlândia, Brazil; 3 Institute of Biotechnology, Federal University of Catalão, Catalão, Brazil; 4 Institute of Biomedical Sciences, Federal University of Uberlândia, Uberlândia, Brazil; 5 Department of Physical Education, Federal University of Sergipe, São Cristóvão, Brazil; 6 Institute of Sport Sciences, The Jerzy Kukuczka Academy of Physical Education, Katowice, Poland

**Keywords:** biomechanics, eccentric tempo, kinematic analysis, para powerlifting, stretch-shortening cycle

## Abstract

**Background:**

Short eccentric phases may enhance concentric performance in resistance exercise, but evidence under competition-specific Paralympic bench press conditions remains limited.

**Objective:**

To investigate the acute effects of eccentric phase duration on concentric bar velocity and power output during competitive Paralympic bench press.

**Methods:**

Thirteen competitive Para Powerlifting athletes (8 women, 5 men) performed single repetitions at 80% of 1RM under six conditions: purely concentric (CONPUR), volitional eccentric without pause (ECCVNP), volitional eccentric with a competition-valid pause (ECCVOL), and controlled eccentric phases of 2 s (ECCFAS), 4 s (ECCMED), and 6 s (ECCSLO), all including a competition-valid pause except CONPUR and ECCVNP. Peak and mean concentric velocity and power were obtained via high-speed video. Differences among conditions were analyzed with one-way repeated-measures ANOVA, Bonferroni-adjusted pairwise comparisons, and partial eta squared (*η*p^2^) as effect size.

**Results:**

Condition significantly affected peak velocity (*p* < 0.001, *η*p^2^ = 0.437), mean velocity (*p* < 0.001, *η*p^2^ = 0.522), peak power output (*p* = 0.001, *η*p^2^ = 0.379), and mean power output (*p* < 0.001, *η*p^2^ = 0.434). ECCVNP produced the highest velocity and power values, while ECCSLO produced the lowest. Eccentric duration differed significantly across eccentric conditions (*p* < 0.001, *η*p^2^ = 0.988), while pause duration did not (*p* = 0.140, *η*p^2^ = 0.139).

**Conclusion:**

Faster eccentric actions, particularly volitional tempos under 4s, were associated with greater concentric velocity and power, whereas prolonged eccentric durations reduced concentric performance in competitive Paralympic bench press.

## 1 Introduction

The bench press is widely used in strength training, serving as both an auxiliary exercise and a tool for performance assessment across various sports disciplines (1–4). In Para Powerlifting, athletes with physical impairments perform the bench press with a maximal load for a single repetition. Unlike non-competitive settings, competitive execution is governed by strict technical rules, including controlled bar descent, mandatory chest contact, a required pause, and simultaneous full elbow extension at the end of the movement (5). These execution requirements can substantially affect barbell kinematics and may influence acute performance outcomes.

The eccentric phase plays an essential role in mechanical efficiency as well as neuromuscular effectiveness during subsequent concentric actions (6, 7). Shorter eccentric durations are generally associated with greater concentric velocity and power output, whereas longer eccentric durations can reduce subsequent concentric performance (8–12). The greater concentric performance observed following faster eccentric actions may be attributed to enhanced utilization of the stretch-shortening cycle (SSC), including more effective storage and reutilization of elastic energy, as well as greater recruitment of high-threshold motor units, particularly those associated with type IIa and IIx muscle fibers (13–15).

Although the effects of eccentric duration on concentric performance have been extensively studied, the transition between the eccentric and concentric phases may be particularly relevant in competitive Para Powerlifting. During a rapid eccentric-to-concentric transition, the preceding eccentric action may contribute to subsequent concentric performance through mechanisms associated with the stretch-shortening cycle (SSC). In contrast, imposing a pause between the eccentric and concentric phases has been shown to reduce the performance augmentation associated with the preceding stretch, suggesting that the timing of the transition may influence the extent to which the SSC contributes to subsequent force production (16). Thus, the mandatory pause in Para Powerlifting may alter the mechanical and neuromuscular conditions under which eccentric tempo influences concentric performance.

Despite its practical relevance for Para Powerlifting coaches and athletes, little is known about how different eccentric durations affect concentric performance under competition-specific conditions. Previous studies examining eccentric tempo have predominantly been conducted in conventional “touch and go” bench press using protocols that do not reproduce the technical requirements of competitive Para Powerlifting (8–12). Consequently, it remains unclear whether the tempo-related patterns reported in conventional bench press generalize to competition-valid execution in athletes with physical impairments. Furthermore, studies examining the acute effects of eccentric tempo manipulation in competitive Paralympic athletes are scarce. Therefore, the present study extends previous research on eccentric tempo by examining concentric bar velocity and power across different eccentric durations under competition-specific Paralympic bench press conditions, including a competition-valid pause.

Therefore, this study aimed to analyze the effect of varying eccentric phase durations on bar velocity and power output during the concentric phase of the competitive Paralympic bench press. Based on previous findings, it was hypothesized that shorter eccentric durations would be associated with greater concentric velocity and power, whereas longer eccentric durations would result in lower concentric performance under competition-specific conditions.

## 2 Materials and methods

### 2.1 Participants

A convenience sample of 13 competitive Para Powerlifting athletes (8 women and 5 men) from Uberlândia, Brazil, voluntarily participated in this study. All 13 athletes met the predefined inclusion criteria, and no participant was subsequently excluded from the study or analysis. The use of convenience sampling was necessary due to the restricted population of athletes meeting the inclusion criteria, the high level of competitive specialization, and the practical challenges involved in recruiting and evaluating elite Paralympic athletes. Consequently, all eligible and available athletes were invited to participate. All participants had at least 3 years of training experience in Paralympic bench press and were actively competing at the time of data collection. Inclusion criteria were: (a) current classification in official Paralympic competitions; and (b) absence of acute musculoskeletal injuries in the upper limbs or trunk. Participants presented a range of impairments, including spinal cord injury, poliomyelitis sequelae, myelomeningocele, and lower limb injuries. Unlike many Paralympic sports that use multiple sport classes to group athletes according to the impact of impairment on sport-specific activities, Para Powerlifting has a single Sport Class for eligible athletes. Once athletes meet the minimum impairment criteria, competition is divided by sex and body mass categories rather than by different functional sport classes (17). Athletes with progressive neuromuscular disorders or recent surgical procedures (< 6 months) were excluded.

Notably, three participants were preparing for the 2024 Paralympic Games, and two of them subsequently won Paralympic medals. The study was conducted in accordance with the principles of the Declaration of Helsinki and the ethical standards for sport and exercise science research (18). The study was approved by the Human Research Ethics Committee of the Federal University of Uberlandia (CAAE: 44681920.8.0000.5152), and all participants provided written informed consent prior to participation. Participant characteristics are presented in Table 1.

**Table 1:** Participant characteristics (mean ± SD).

Variable	Female (*n* = 8)	Male (*n* = 5)
Age (years)	28.3 ± 6.5	32.6 ± 7.9
Body mass (kg)	65.4 ± 17.3	87.1 ± 17.2
Relative strength (1RM/body mass)	1.4 ± 0.5	1.5 ± 0.3
Training experience (years) Body mass category	6.9 ± 3.56 41 kg; 45 kg; 55 kg; 73 kg; 79 kg; 86 kg	11.0 ± 3.94 59 kg; 88 kg; 107 kg

### 2.2 Experimental design

A repeated-measures crossover design was implemented. All participants completed the bench press exercise under six cadence conditions in a single testing session. The order of conditions was randomized using a computer-generated sequence (R software, v.4.2), ensuring that no participant initiated trials with the same cadence. A minimum rest interval of 3 min and a maximum of 5 min was allowed between sets to prevent residual fatigue.

Each condition was performed with 80% of the athlete's 1RM, obtained from official competition within 2–10 days before testing. Athletes abstained from resistance training for at least 24 h prior to the session.

### 2.3 Exercise conditions

Participants performed sets under six conditions: CONPUR (0/0/X), purely concentric from the bar resting position with maximal concentric effort; ECCVNP (V/0/X), volitional eccentric without a pause and maximal concentric effort; and ECCVOL (V/P/X), volitional eccentric with a competition-valid pause and maximal concentric effort. The eccentric controlled conditions were ECCFAS (2/P/X), ECCMED (4/P/X), and ECCSLO (6/P/X), corresponding to 2 s, 4 s and 6 s eccentric phases, respectively, each followed by a competition-valid pause and maximal concentric effort (Figure 1).

**Figure 1:**
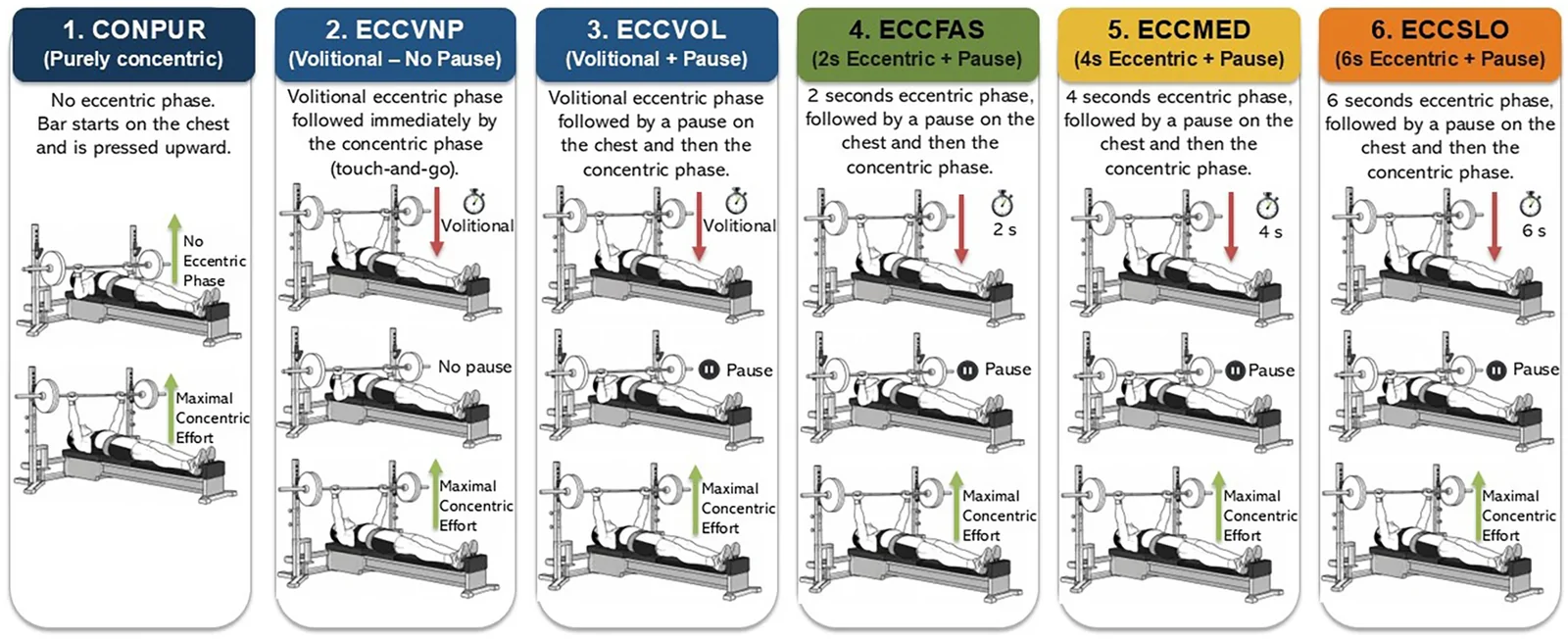
Illustrative representation of the experimental conditions evaluated in the study. The figure was developed using AI ChatGPT (OpenAI; GPT-5.6 Luna) and subsequently edited, organized, and annotated by the authors.

A metronome (Pro Metronome, EUM) was used to standardize non-volitional eccentric tempos. The pause duration was adjudicated by a certified national referee following Para Powerlifting rules.

### 2.4 Warm-up and familiarization

Participants completed a standardized warm-up consisting of 15, 10, 5, and 3 repetitions at 20%, 40%, 60% and 70% of their 1RM, respectively, at volitional tempo, with a pause and a 2-minute interval between sets. Subsequently, familiarization with non-volitional cadences (2 s, 4 s, 6 s) consisted of two single repetitions at 60% 1RM per cadence and 2-minute rest interval between sets. A 5-minute rest interval was provided between the end of the familiarization procedures and the beginning of the experimental trials to avoid residual effects on performance.

All participants had previous training experience with tempo-controlled eccentric repetitions, including both fast and slow eccentric phases, which minimized potential learning effects associated with adopting controlled cadences during testing. No familiarization repetitions were included in the analyses.

### 2.5 Data measurement

Two single repetitions were performed at 80% of 1RM for each cadence condition, with a 3–5 min rest interval between sets. An intensity of 80% of 1RM was chosen because the participants lacked familiarity with prolonged eccentric phases combined with a pause, particularly at higher intensities, which could compromise both data reliability and participant safety. Moreover, ≥80% of 1RM represents a high-load condition that allows the expression and assessment of the distinct phases of the concentric movement through the barbell velocity profile (19), while still permitting successful execution across the different eccentric tempo conditions. Only the repetition with eccentric duration closest to the target cadence was used for statistical analysis. When both repetitions met the cadence criteria, the first valid repetition was selected according to a prespecified procedure. Averaging the two repetitions was not performed because differences in the achieved eccentric durations between repetitions could result in an averaged value that did not accurately represent the prescribed experimental condition. Thus, selecting the repetition closest to the target cadence was considered more appropriate for preserving the fidelity of the eccentric tempo manipulation, although this approach does not allow averaging to reduce trial-to-trial measurement variability.

Barbell trajectory was acquired using a digital camera (HD 2D, 240 fps) positioned perpendicular to the sagittal plane, 1.50 m from the right bar support and 0.92 m above the ground. A reflective LED marker was attached to the lateral surface of the bar on the dominant side of each participant to facilitate tracking of the barbell's movement (20).

### 2.6 Variables

Barbell kinematics were assessed using the tracking tool in Kinovea software (version 0.9.5) (21, 22), which has demonstrated acceptable validity and reliability for assessing barbell kinematics (23), including agreement between evaluators with different levels of experience (24). Mean and peak velocity were derived from the barbell's vertical displacement and the corresponding time intervals. Instantaneous velocity was calculated using the following formula:

$$v_{i}=\frac{x_{\left\{i+1\right\}}-x_{\left\{i-1\right\}}}{t_{\left\{i+1\right\}}-t_{\left\{i-1\right\}}}$$

where *v_i_* is instantaneous vertical velocity, *x*_*i*+1_ and *x*_*i*−1_ are the vertical displacement values at the subsequent and preceding time points, respectively, and *t*_*i*+1_ and *t*_*i*−1_ are the corresponding time points.

Mean and peak concentric power output were calculated using the following formula:

P(t)=m×[g+a(t)]×v(t)

where *P(t)* is instantaneous power, *m* is the total mass of the barbell and plates, *g* is gravitational acceleration, *a(t)* is instantaneous vertical acceleration, and *v(t)* is instantaneous vertical velocity.

Calibration was performed using an object of known dimensions placed in the same plane as the marker.

### 2.7 Statistical analysis

All statistical analyses were performed using MATLAB R2025b (MathWorks, Natick, MA, USA). Data are presented as mean ± standard deviation (SD). Normality was assessed using the Shapiro–Wilk test. Differences in peak velocity, mean velocity, peak power, and mean power across the six experimental conditions were assessed using one-way repeated-measures ANOVA. Sphericity was assessed using Mauchly's test, and the Greenhouse–Geisser correction was applied when necessary. Partial eta squared (*η*^2^p) was used as a measure of effect size. When a significant main effect was observed, paired comparisons with Bonferroni adjustment were performed, with mean differences, 95% confidence intervals, and Cohen's *d*z reported.

Eccentric phase duration was analyzed across the five conditions involving an eccentric phase (ECCVNP, ECCVOL, ECCSLO, ECCMED, and ECCFAS), using the same statistical procedures. Pause duration was analyzed across ECCVOL, ECCSLO, ECCMED, and ECCFAS, as no prescribed pause was present in CONPUR or ECCVNP. All tests were two-tailed, and statistical significance was set at *p* < 0.05.

## 3 Results

### 3.1 Achieved eccentric and pause durations

The achieved eccentric and pause durations for each experimental condition are presented in Table 2. Eccentric duration progressively increased across the prescribed eccentric conditions, whereas pause duration remained relatively similar across conditions in which a pause was performed.

**Table 2:** Achieved eccentric and pause durations across experimental conditions.

Condition	Eccentric duration (s)	Pause duration (s)
CONPUR	—	—
ECCVNP	0.96 ± 0.22	—
ECCVOL	1.19 ± 0.28	0.56 ± 0.15
ECCFAS	2.19 ± 0.25	0.61 ± 0.16
ECCMED	3.90 ± 0.20	0.70 ± 0.15
ECCSLO	5.86 ± 0.32	0.65 ± 0.21

Values are presented as mean ± SD. Em dashes indicate that the corresponding phase was not applicable to that condition (CONPUR has no eccentric phase; CONPUR and ECCVNP were performed without a pause). CONPUR = purely concentric; ECCVN*P* = volitional eccentric without pause; ECCVOL = volitional eccentric with competition-valid pause; ECCFAS = 2-s eccentric phase; ECCMED = 4-s eccentric phase; ECCSLO = 6-s eccentric phase.

Eccentric duration differed significantly among experimental conditions [F(4,48) = 1001.43, *p* < 0.001, *η*p^2^ = 0.988]. Mauchly's test indicated that the assumption of sphericity was not violated (W = 0.493, *p* = 0.598). Pairwise comparisons showed significant differences across all pairwise comparisons after Bonferroni correction (all *p* ≤ 0.047). The smallest absolute difference was observed between ECCVNP and ECCVOL [mean difference = −0.239 s, 95% CI (−0.390, −0.089), Bonferroni-adjusted *p* = 0.047, Cohen's *d*z = −0.961], whereas the largest absolute difference was observed between ECCVNP and ECCSLO [mean difference = −4.904 s, 95% CI (−5.114, −4.694), Bonferroni-adjusted *p* < 0.001, Cohen's *d*z = −14.100]. Detailed pairwise comparisons are presented in Supplementary Table S1.

Pause duration did not differ significantly among eccentric conditions [*F*(3,36) = 1.94, *p* = 0.140, *η*^2*p*^ = 0.139]. Mauchly's test indicated that the assumption of sphericity was not violated (*W* = 0.506, *p* = 0.200). Mean pause durations were 0.556 ± 0.147 s for ECCVOL, 0.654 ± 0.208 s for ECCSLO, 0.695 ± 0.152 s for ECCMED, and 0.612 ± 0.162 s for ECCFAS.

### 3.2 Achieved concentric performance conditions

The experimental condition significantly affected all concentric performance variables (Table 3). Peak velocity, mean velocity, peak power, and mean power differed significantly across conditions, with large effect sizes (*η*p^2^ = 0.379–0.522). Descriptively, ECCVNP showed the highest mean values for peak velocity, mean velocity, and mean power, whereas peak power was highest in ECCVNP, followed by ECCVOL. In contrast, ECCSLO and ECCMED generally showed the lowest velocity and power values.

**Table 3:** Concentric velocity and power output across experimental conditions.

Variable	CONPUR	ECCVNP	ECCVOL	ECCFAS	ECCMED	ECCSLO	F (df1, df2)	p	*η*p^2^
Peak velocity (m/s)	0.491 ± 0.168	0.546 ± 0.087	0.523 ± 0.104	0.484 ± 0.121	0.420 ± 0.105	0.404 ± 0.084	*9.31* (*5, 60)*	<0.001	0.437
Mean velocity (m/s)	0.314 ± 0.122	0.387 ± 0.068	0.352 ± 0.077	0.332 ± 0.091	0.292 ± 0.070	0.281 ± 0.067	*13.10* (*2.38, 28.56)*	<0.001	0.522
Peak power output (W)	409.4 ± 144.7	484.9 ± 175.3	457.3 ± 174.6	417.9 ± 153.0	363.6 ± 155.7	338.6 ± 124.0	*7.31* (*2.49, 29.87)*	0.001	0.379
Mean power output (W)	250.4 ± 95.3	315.1 ± 99.3	289.7 ± 106.5	265.7 ± 84.2	236.4 ± 79.4	225.7 ± 76.1	*9.19* (*2.06, 24.72)*	<0.001	0.434

Values are presented as mean ± SD. F ratios reflect one-way repeated-measures ANOVA with condition as the within-subject factor; degrees of freedom were adjusted using the Greenhouse–Geisser correction when the assumption of sphericity was violated according to Mauchly's test. ηp^2^ = partial eta squared. CONPUR = purely concentric; ECCVNP = volitional eccentric without pause; ECCVOL = volitional eccentric with competition-valid pause; ECCSLO = 6-s eccentric phase; ECCMED = 4-s eccentric phase; ECCFAS = 2-s eccentric phase.

In summary, across the pairwise comparisons presented below, the most consistent pattern was that the two fastest eccentric conditions (ECCVNP and ECCVOL) produced significantly higher peak and mean velocity and power than the two most prolonged conditions (ECCMED and ECCSLO), whereas CONPUR and the ECCVNP vs. ECCVOL comparison did not differ significantly.

### 3.3 Concentric velocity

Bonferroni-adjusted pairwise comparisons indicated significant differences among several experimental conditions for both peak and mean concentric velocity. For peak velocity, ECCVNP and ECCVOL were significantly higher than ECCSLO and ECCMED, while ECCSLO was significantly lower than ECCFAS. For mean velocity, ECCVNP and ECCVOL were significantly higher than ECCSLO and ECCMED, and ECCVNP was also significantly higher than ECCFAS. Additionally, ECCSLO showed lower mean velocity than ECCFAS (Table 4).

**Table 4:** Significant pairwise comparisons of peak and mean velocity following Bonferroni correction.

Variable	Comparison	Mean difference	95% CI	p (Bonferroni)	Cohen's dz
Mean velocity	ECCVNP vs. ECCSLO	0.106	0.081–0.131	<0.001	2.580
	ECCVNP vs. ECCMED	0.094	0.078–0.111	<0.001	3.501
	ECCVNP vs. ECCFAS	0.055	0.027–0.083	0.015	1.201
	ECCVOL vs. ECCSLO	0.071	0.044–0.099	0.002	1.559
	ECCVOL vs. ECCMED	0.059	0.039–0.080	<0.001	1.765
	ECCSLO vs. ECCFAS	−0.051	−0.078 - −0.024	0.020	−1.149
Peak velocity	ECCVNP vs. ECCSLO	0.141	0.103–0.180	<0.001	2.223
	ECCVNP vs. ECCMED	0.125	0.086–0.165	<0.001	1.906
	ECCVOL vs. ECCSLO	0.119	0.075–0.162	<0.001	1.660
	ECCVOL vs. ECCMED	0.103	0.064–0.142	0.001	1.587
	ECCSLO vs. ECCFAS	−0.079	−0.122 - −0.037	0.024	−1.123

CI, confidence interval. Only statistically significant comparisons (Bonferroni-adjusted *p* < .05) are shown; ECCVNP = volitional eccentric without pause; ECCVOL = volitional eccentric with competition-valid pause; ECCSLO = 6-s eccentric phase; ECCMED = 4-s eccentric phase; ECCFAS = 2-s eccentric phase.

### 3.4 Concentric power output

Bonferroni-adjusted pairwise comparisons also revealed significant differences among several experimental conditions for peak and mean concentric power. For peak power, ECCVNP and ECCVOL were significantly higher than ECCSLO and ECCMED. For mean power, ECCVNP and ECCVOL were significantly higher than ECCSLO and ECCMED, while ECCVNP also showed higher values than ECCFAS. In addition, ECCSLO showed lower mean power than ECCFAS (Table 5).

**Table 5:** Significant pairwise comparisons of peak and mean power output following Bonferroni correction.

Variable	Comparison	Mean difference	95% CI	p (Bonferroni)	Cohen's dz
Mean power output	ECCVNP vs. ECCSLO	89.3	62.9 to 115.8	<0.001	2.042
	ECCVNP vs. ECCMED	78.7	58.3 to 99.1	<0.001	2.327
	ECCVNP vs. ECCFAS	49.3	20.6 to 78.1	0.043	1.036
	ECCVOL vs. ECCSLO	64.0	36.1 to 91.9	0.005	1.384
	ECCVOL vs. ECCMED	53.4	31.0 to 75.7	0.003	1.443
	ECCSLO vs. ECCFAS	−40.0	−63.5 to −16.4	0.046	−1.025
Peak power output	ECCVNP vs. ECCSLO	146.3	100.0 to 192.6	<0.001	1.909
	ECCVNP vs. ECCMED	121.3	73.7 to 168.9	0.002	1.541
	ECCVOL vs. ECCSLO	118.7	69.3 to 168.2	0.003	1.451
	ECCVOL vs. ECCMED	93.7	56.1 to 131.4	0.002	1.506

CI, confidence interval. Power output values are expressed in watts (W). Only statistically significant comparisons (Bonferroni-adjusted *p* < .05) are shown; ECCVNP = volitional eccentric without pause; ECCVOL = volitional eccentric with competition-valid pause; ECCSLO = 6-s eccentric phase; ECCMED = 4-s eccentric phase; ECCFAS = 2-s eccentric phase.

## 4 Discussion

The main finding of this study was that prolonging the eccentric phase to ≥4 s (ECCMED, ECCSLO) reduced concentric bar velocity and power output relative to the two fastest eccentric tempos (ECCVNP, ECCVOL), with large and highly consistent effect sizes across all four outcome variables (Cohen's *dz* = 1.45–3.50). This was the only pattern confirmed across peak velocity, mean velocity, peak power output, and mean power output alike. These findings should be interpreted within the tested intensity of 80% of 1RM, which represents a high load but may not fully reflect the loads used during maximal competition attempts. Because concentric velocity decreases as relative load increases in Para Powerlifting, the performance cost associated with prolonged eccentric durations may differ at higher competitive loads. However, several comparisons that might intuitively be expected to differ did not reach statistical significance; this includes every pairwise comparison involving the purely concentric condition (CONPUR) and the comparison between the two fastest eccentric tempos (ECCVNP vs. ECCVOL). These results partially support the initial hypothesis that shorter eccentric durations were associated with better concentric outcomes, but the statistically confirmed comparisons are more limited than descriptive percentage differences alone would suggest. Therefore, interpretation of the findings should consider both statistical significance and the magnitude of the observed effects (25).

The heterogeneity of impairment types in the present sample should also be considered when interpreting the findings. Although all participants competed within the same Sport Class, differences in impairment characteristics may contribute to interindividual variability in movement mechanics and performance. Previous research in Para Powerlifting has reported differences in strength and competitive performance according to impairment characteristics and sport classification-related factors (26). In addition, differences in body mass category and strength level may contribute to variability in absolute performance. Nevertheless, the repeated measures design of the present study allowed each athlete to perform all experimental conditions, such that the primary comparisons were made within individuals.

Consistent with this overall pattern, both fast eccentric strategies, performed with no pause (ECCVNP, ≈0.96 s) and under competition constraints (ECCVOL, ≈1.19 s), produced significantly higher peak velocity, mean velocity, peak power, and mean power than the two most prolonged tempos, ECCMED (≈3.90 s) and ECCSLO (≈5.86 s), with large effect sizes throughout (mean differences 0.059–0.141 m/s and 53.4–146.3 W; dz = 1.45–3.50). This agrees with previous non-competitive bench press studies reporting progressively lower concentric velocity, power, and 1RM as eccentric tempo lengthens from approximately 2 to 6 s (8, 27, 28). The 2-s condition (ECCFAS) occupied an intermediate, statistically ambiguous position: it was significantly higher than ECCSLO for peak velocity, mean velocity, and mean power (dz = −1.02 to −1.15), but not for peak power, and it did not differ significantly from ECCMED for any variable. ECCFAS also did not differ significantly from ECCVNP or ECCVOL, except that ECCVNP was significantly higher than ECCFAS for mean velocity and mean power specifically (dz = 1.04–1.20). These findings provide some context for the previously reported range of approximately 1.0–1.4 s associated with optimal concentric velocity (9). In the present competition-constrained protocol, the 2-s eccentric phase did not reliably differ from the faster tempos, suggesting that a short prolongation beyond the athlete's self-selected pace may not necessarily result in a clear performance reduction when the mandatory competition pause is also present.

Rather than indicating a single durational threshold, the data therefore suggest a graded pattern in which performance costs became statistically detectable once eccentric duration reached approximately 4 s, whereas a 2-s eccentric phase produced intermediate outcomes that were not consistently distinguishable from either the fast or the prolonged conditions.

Comparing the touch-and-go condition (ECCVNP) with the purely concentric condition (CONPUR) provides additional context. ECCVNP produced approximately 23%–26% higher mean velocity and power output, and 11%–18% higher peak velocity and peak power output, than CONPUR. This is consistent with previous reports of greater 1RM performance, velocity and power output with touch-and-go compared with purely concentric techniques (7, 16, 29, 30). These findings suggest that the ability to transition from the eccentric to the concentric phase may contribute to greater concentric output.

Although CONPUR could not be statistically distinguished from any condition, it showed descriptively lower velocity and power output than the faster eccentric conditions and relatively high between-participant variability, particularly for peak velocity (SD = 0.168 m/s; Table 3). This greater variability may reflect the distinct movement strategy of initiating the lift from a dead stop without a preceding eccentric phase. Because the load was fixed at 80% of 1RM across all conditions, differences in power output were largely attributable to differences in bar velocity rather than changes in the external load.

Pause duration itself did not differ significantly among the eccentric conditions in which it was performed, indicating that the pause was applied consistently regardless of eccentric tempo. Isolating the specific contribution of the pause is more difficult than isolating that of eccentric duration. The most direct comparison for exploring a potential pause effect was between ECCVNP and ECCVOL, which differed by the presence of the mandatory pause, although their eccentric durations also differed. ECCVNP showed numerically higher velocity and power output than ECCVOL, with differences of approximately 6%–9% across the four outcome variables, but none of these differences reached statistical significance. Previous research using dynamic designs has reported measurable reductions in performance with imposed pauses of comparable duration (16). However, the present data do not provide clear statistical support for a specific effect of the mandatory pause on performance. Nevertheless, even small absolute differences may have practical relevance in high-performance contexts, where marginal changes in performance may influence competitive outcomes (31).

The pattern observed, characterized by large and consistent advantages of shorter eccentric tempos over prolonged tempos (≥4 s), together with numerically higher velocity and power output in the unpaused condition, is compatible with mechanisms involving the stretch-shortening cycle (SSC). Rapid eccentric actions may allow greater utilization of elastic energy during the subsequent concentric phase, whereas longer transition times may reduce this contribution (13, 14, 32–35). However, the present study did not include measures that could directly assess SSC behavior or elastic energy utilization, and the ECCVNP versus ECCVOL comparison, which provides the most direct contrast for examining a potential effect of the pause, did not reach statistical significance. Therefore, the observed differences in bar velocity and power cannot be attributed specifically to elastic energy storage and reutilization. Other mechanisms may also contribute, including neuromuscular potentiation, changes in movement coordination and greater perceived effort or fatigue associated with prolonged eccentric actions (36). Thus, these mechanisms should be considered possible explanations consistent with previous literature rather than effects directly demonstrated by the present study. In particular, the potential role of the SSC and elastic energy in explaining the differences between paused and unpaused conditions remains provisional and requires direct investigation using measures of muscle activation, force production, and elastic behavior.

### 4.1 Practical applications

As a practical application, the best-supported recommendation from these data is to avoid prolonging the eccentric phase to 4 s or beyond during competition, as durations in this range were associated with clear, large, and statistically robust reductions in concentric velocity and power output compared with faster, competition-like tempos. Although the 2-s eccentric condition (ECCFAS) and the self-paced competition condition (ECCVOL) did not differ significantly, absolute differences of approximately 7% for velocity and 9% for power output were observed and may be considered when selecting an eccentric tempo strategy, although these effects were not statistically confirmed.

Deliberately prolonged eccentric tempos (≥4 s) may be more appropriate for training contexts aimed at increasing time under tension or perceived effort than for maximal competition attempts. Training strategies aimed at improving the transition from the eccentric to the concentric phase may be considered; however, this possibility remains speculative and requires direct investigation.

### 4.2 Limitations and future research

Although this study provides important evidence on the influence of eccentric tempo on concentric output under competition-specific conditions, several limitations should be acknowledged. The small sample (n = 13), resulting from the limited availability of eligible competitive Para Powerlifting athletes, may have reduced statistical power and generalizability. No *a priori* sample size calculation was performed because of the restricted pool of active, competition-eligible Para Powerlifting athletes available for recruitment. Consequently, the study may have been underpowered to detect smaller true effects, particularly for comparisons that did not reach statistical significance. The mixed-sex and impairment-heterogeneous sample also prevented separate analyses by sex or impairment type. As testing was conducted in a single session, cumulative fatigue cannot be entirely excluded despite randomized condition order and standardized rest intervals. Findings were obtained at 80% of 1RM and may not fully represent maximal competition loads. Although intra-rater and inter-rater reliability of video digitization was not formally assessed, future studies should report protocol-specific reliability metrics. Finally, the absence of neuromuscular measurements limited direct verification of the physiological mechanisms underlying the observed effects. Future research should use larger, impairment-stratified samples, higher competition-relevant loads, and combined kinematic and neuromuscular measures to confirm and extend these findings.

## 5 Conclusion

In competitive Para Powerlifting, eccentric phase duration had a significant acute effect on concentric performance. Fast, competition-valid eccentric tempos produced higher bar velocity and power output than prolonged eccentric durations of ≥4 s, with large effect sizes. In contrast, the 2-s eccentric condition did not consistently differ from the faster tempos, while the specific effect of the mandatory pause was not statistically confirmed. Therefore, athletes should avoid deliberately prolonging the eccentric phase to ≥4 s during maximal competition attempts, while maintaining the mandatory pause as prescribed.

## Data Availability

The original contributions presented in the study are included in the article/Supplementary Material, further inquiries can be directed to the corresponding author.
