# Supplementary material for: Influence of eccentric tempo on concentric performance in competitive Paralympic bench press
Source: Front Sports Act Living. 2026 Oct 26;8:1963961. doi: 10.3389/fspor.2026.1963961 (PMC13746365; doi:10.3389/fspor.2026.1963961)
Supplement: Supplementary file 1 [file Table1.docx]

**Influence of Eccentric Tempo on Concentric Performance in Competitive Paralympic Bench Press**

**Arthur Luz Santos^1^, Bárbara G. Silva^2^, Silvio S. Santos^2^, Thiago M. Fidale ^1,3^, Frederico B. Lizardo^1,4^, Weverton Santos^1^, Felipe J. Aidar^5^, Michal Wilk^6^, Adriano A. Pereira^1^.**

^1^ Postgraduate Program in Biomedical Engineering, Federal University of Uberlândia; Uberlândia, MG, Brazil.

^2^ Faculty of Physical Education and Physiotherapy, Federal University of Uberlândia; Uberlândia, MG, Brazil.

^3^ Institute of Biotechnology - Federal University of Catalão; Catalão, GO, Brazil

^4^ Institute of Biomedical Sciences - Federal University of Uberlândia; Uberlândia, MG, Brazil.

^5^ Department of Physical Education, Federal University of Sergipe, São Cristóvão, Brazil;

^6^ Institute of Sport Sciences, The Jerzy Kukuczka Academy of Physical Education, Katowice, Poland;

**Supplementary Table S1.** Pairwise comparisons of achieved eccentric and pause durations following Bonferroni correction.

| **Comparison** | **Mean difference (s)** | **95% CI** | **p (Bonferroni)** | **Cohen's dz** |
| --- | --- | --- | --- | --- |
| ECCVNP vs. ECCVOL | −0.239 | −0.390 to −0.089 | 0.047 | −0.961 |
| ECCVNP vs. ECCSLO | −4.904 | −5.114 to −4.694 | <0.001 | −14.100 |
| ECCVNP vs. ECCMED | −2.948 | −3.100 to −2.796 | <0.001 | −11.704 |
| ECCVNP vs. ECCFAS | −1.230 | −1.464 to −0.996 | <0.001 | −3.181 |
| ECCVOL vs. ECCSLO | −4.665 | −4.826 to −4.504 | <0.001 | −17.507 |
| ECCVOL vs. ECCMED | −2.709 | −2.899 to −2.518 | <0.001 | −8.584 |
| ECCVOL vs. ECCFAS | −0.991 | −1.195 to −0.787 | <0.001 | −2.933 |
| ECCSLO vs. ECCMED | 1.956 | 1.744 to 2.168 | <0.001 | 5.582 |
| ECCSLO vs. ECCFAS | 3.674 | 3.425 to 3.923 | <0.001 | 8.918 |
| ECCMED vs. ECCFAS | 1.718 | 1.503 to 1.932 | <0.001 | 4.839 |

Note. Values are presented as mean differences. All pairwise comparisons were statistically significant after Bonferroni correction. CI = confidence interval; ECCVNP = volitional eccentric without pause; ECCVOL = volitional eccentric with competition-valid pause; ECCSLO = 6-s eccentric phase; ECCMED = 4-s eccentric phase; ECCFAS = 2-s eccentric phase.
